# Supplementary material for: Knowledge, Attitude and Practice of the Community General Practice Teams on Dysphagia Complicated with Aspiration Pneumonia after Stroke
Source: Healthcare (Basel). 2023 Nov 30;11(23):3073. doi: 10.3390/healthcare11233073 (PMC10706243; doi:10.3390/healthcare11233073)
Supplement: Supplementary file 1 [file healthcare-11-03073-s001.zip › healthcare-2602684-supplementary.pdf]

healthcare

MDPI

Article

Knowledge, Attitude, and Practice of the Community General Practice Teams on Dysphagia Complicated with Aspiration Pneumonia after Stroke

Daikun He [1,2,3], Xueting Shen [1], Lina Wang [2] and Zhigang Pan [1]*

[1]Department of General Practice, Zhongshan Hospital, Fudan University, Shanghai 200032, China;

[2]Department of General Practice, Jinshan Hospital, Fudan University, Shanghai 201508, China;

[3]Center of Emergency and Critical Care Medicine, Jinshan Hospital, Fudan University, Shanghai 201508, China

* Correspondence: author Zhigang Pan Department of General Practice, Zhongshan Hospital, Fudan University, Shanghai 200032, China; Email: zhigang_pan@163.com; Tel: +86-18616881251

1. Supplementary Materials

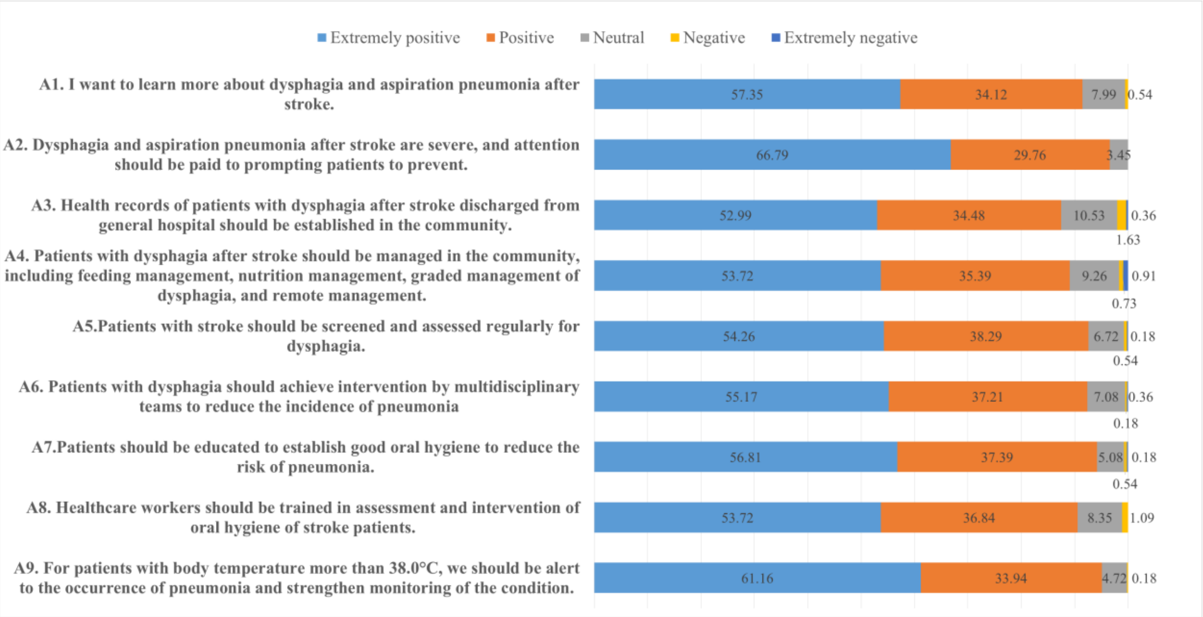

Supplementary Figure S1. The distribution of the “attitude” dimension.

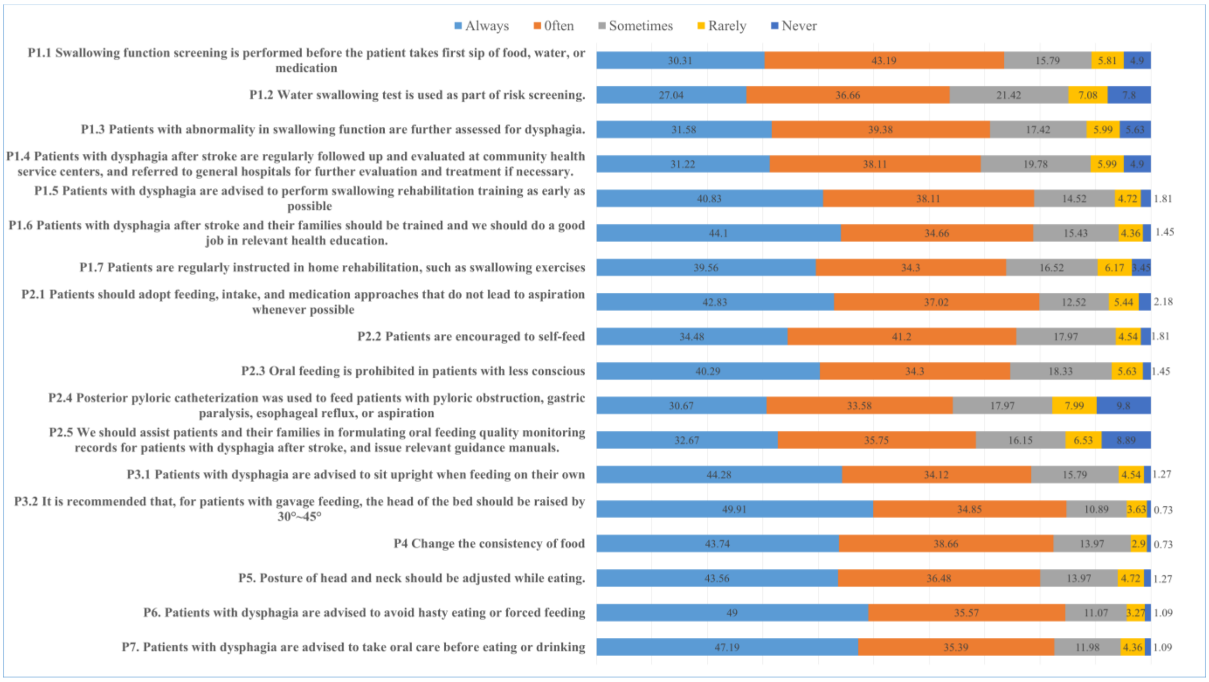

Supplementary Figure S2. The distribution of the "practice" dimension.

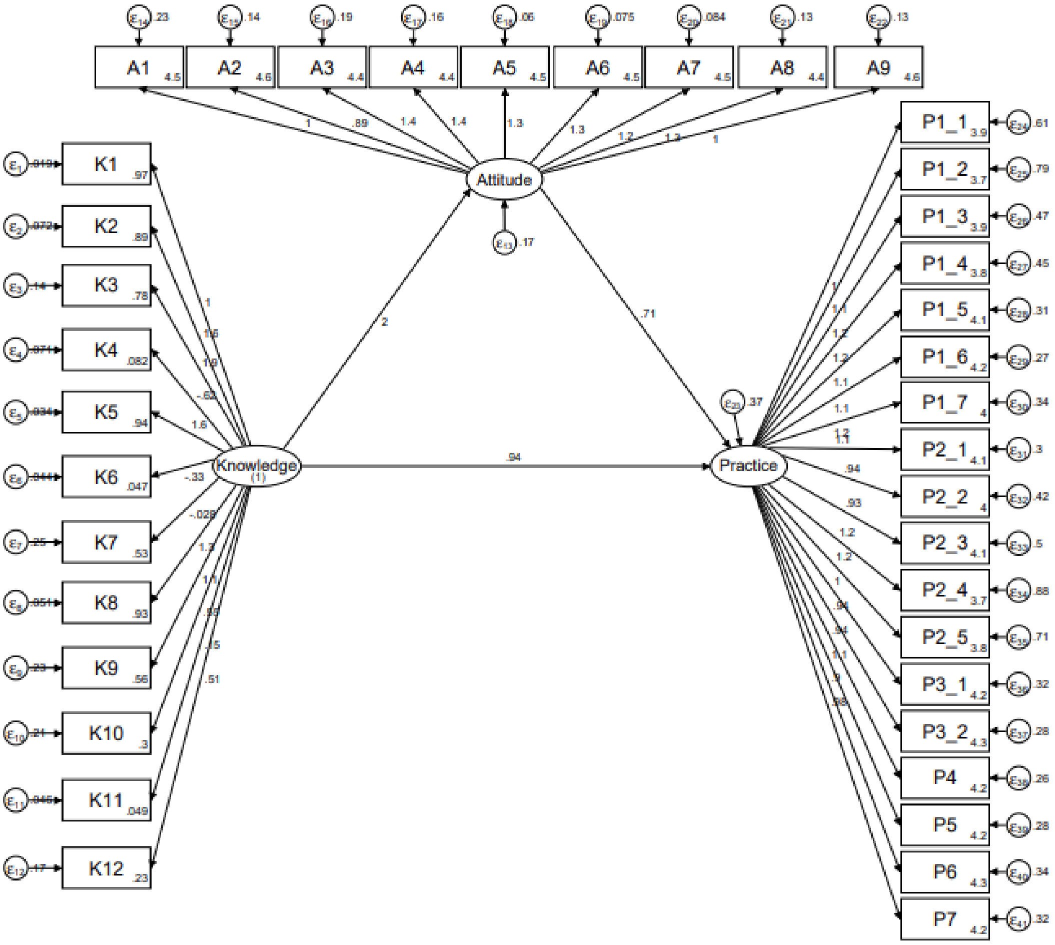

Supplementary Figure S3. Results of structural equation model (SEM) for the association among Knowledge, Attitude, and Practice.

Supplementary Table S1. Knowledge dimension.

Knowledge, n (%)	Participants (n=551)	
	True	False/unclear
1. Dysphagia is one of the most common clinical complications after stroke, greatly increasing the risk of death and poor prognosis.	535 (97.10)	16 (2.90)
2. Dysphagia is a major risk factor for pneumonia after stroke, with aspiration occurring in more than 40% of patients with dysphagia.	491 (89.11)	60 (10.89)
3. International scales for stroke-related pneumonia include acute ischemic stroke-associated pneumonia score (AIS-APS) and spontaneous intracerebral hemorrhage-associated pneumonia score (ICH-APS).	428 (77.68)	123 (22.32)
4. Stroke-associated pneumonia is new pneumonia that develops within 14 days of onset in nonmechanically ventilated stroke patients. (False)	45 (8.17)	506 (91.83)
5. Early assessment, screening, and rehabilitation of swallowing function after acute stroke can reduce the incidence of pneumonia.	517 (93.83)	34 (6.17)
6. Anti-infective therapy should be initiated within 4 hours in all patients with suspected or confirmed stroke-associated pneumonia if patients were complicated by sepsis or septic shock. (False)	26 (4.72)	525 (95.28)
7. For stroke patients, anti-infective drugs can be used prophylactically. (False)	291 (52.81)	260 (47.19)
8. Raising the head of the bed by 30°-45° is an effective measure to prevent stroke-associated pneumonia	511 (92.74)	40 (7.26)
9. Dysphagia and aspiration after stroke can cause a variety of complications, including:	309 (56.08)	242 (43.92)
10. Aspiration and pneumonia are more likely to occur under combined factors.	167 (30.31)	384 (69.69)
11. To avoid stroke-associated pneumonia, which of the following drugs should be minimized?	27 (4.90)	524 (95.10)
12. For aspiration pneumonia, anti-infective therapy should be covered.	124 (22.50)	427 (77.50)

Disclaimer/Publisher's Note: The statements, opinions, and data contained in all publications are solely those of the individual author(s) and contributor(s) and not of MDPI and/or the editor(s). MDPI and/or the editor(s) disclaim responsibility for any injury to people or property resulting from any ideas, methods, instructions, or products referred to in the content.
